# Influencing Factors in Corneal Densitometry Recovery After Accelerated Cross-Linking for Keratoconus

**DOI:** 10.1155/joph/9490950

**Published:** 2025-02-28

**Authors:** Kuan-I Huang, Cyuan-Yi Yeh, Chao-Chien Hu, Sheng-Fu Cheng

**Affiliations:** ^1^Department of Ophthalmology, Shin Kong Wu Ho-Su Memorial Hospital, Taipei, Taiwan; ^2^School of Medicine, Fu Jen Catholic University, New Taipei City, Taiwan

**Keywords:** 0.25% riboflavin, corneal densitometry, cross-linking (CXL), demarcation line depth (DLD), keratoconus

## Abstract

This study examines corneal densitometry recovery and influencing factors following accelerated corneal cross-linking (CXL) for progressive keratoconus. Corneal densitometry, measured using Scheimpflug tomography, provides an objective assessment of corneal clarity, especially in tracking the resolution of postoperative haze. We conducted a retrospective case-control analysis of 24 patients (31 eyes) who underwent CXL with 0.25% riboflavin and 18 mW/cm^2^ irradiation between 2021 and 2023. Variables included patient age, maximum keratometry (*K*_max_), central corneal thickness (CCT), and demarcation line depth (DLD), defined as the depth of the CXL region. Results revealed a significant increase in densitometry values across most corneal zones at 1-month postoperation, followed by a gradual return to baseline by 12 months. Notably, younger patients exhibited a faster recovery, with mean densitometry values returning to baseline in 11.4 months compared to 14.9 months in older patients (*p*=0.02). Similarly, corneas with deeper DLDs demonstrated faster densitometry recovery, suggesting a potentially more efficient corneal remodeling process. Additional analysis indicated a trend toward higher densitometry values in shallower DLDs at 1 month, although this difference was not statistically significant. These findings support the use of densitometry as a reliable measure of post-CXL healing. While DLD depth and patient age were associated with a faster recovery, they did not directly predict final corneal clarity. Our study suggests that factors such as age and DLD depth should be considered in patient prognosis, although further research is needed to confirm these findings across varying CXL protocols.

## 1. Introduction

Corneal cross-linking (CXL) has become a well-established procedure for halting the progression of keratoconus and other corneal ectatic disorders [1–3]. Following CXL, the cornea undergoes microstructural changes including keratocyte loss and reorganization of collagen lamellae which may result in clinically observable haze. Following CXL, a recovery period is necessary during which the corneal transparency, or clarity, gradually returns. One important parameter used to assess this recovery is corneal densitometry, which provides a quantitative measure of corneal opacity. By evaluating light-scattering properties via Scheimpflug tomography, densitometry offers an objective method to monitor the healing process [4–11]. Previous studies have demonstrated that densitometry values initially increase after CXL but typically decrease over time as the cornea recovers [7].

Standard CXL procedure typically employs a 0.1% riboflavin solution [1]. However, recent studies have begun to investigate the efficacy of higher concentrations of riboflavin, such as 0.25% [12–15]. These higher concentrations have been shown to enhance riboflavin penetration and UVA absorption within the cornea, improving the depth and uniformity of cross-linking [12, 13], and potentially leading to a more effective treatment outcome by enhancing optical properties, such as light absorption [14]. Further investigation is needed to determine whether this higher concentration impacts the densitometry recovery time and to validate the safety of using this concentration in CXL. In addition to riboflavin concentration, several other factors may influence the recovery of corneal densitometry post-CXL. Demarcation line depth (DLD) indicates the depth of the cross-linked region within the cornea. Deeper DLDs are generally associated with more extensive cross-linking, which has been linked to better biomechanical strengthening and clinical outcomes [16, 17]; however, the correlation between DLD and the recovery of corneal transparency remains unclear. Clarifying this relationship could provide valuable insight into optimizing treatment depth for effective CXL. Other variables, such as patient demographics and preoperative corneal characteristics such as age, gender, maximum keratometry (*K*_max_), and central corneal thickness (CCT), may also impact the corneal healing process. While younger age is often associated with quicker recovery periods, emerging evidence suggests that this correlation may not be consistently observed [18–20]. Similarly, baseline *K*_max_ and CCT could influence the cornea's response to CXL, potentially affecting the densitometry recovery timeline [18, 20].

This study aims to assess the impact of 0.25% riboflavin concentration on the time to recover corneal clarity as measured by densitometry and to explore the influence of DLD, age, gender, *K*_max_, and CCT on this recovery process. By analyzing these factors, we seek to provide a comprehensive evaluation of the efficacy and safety of higher riboflavin concentrations in CXL and to identify potential predictors of recovery time.

## 2. Methods

### 2.1. Study Design

This study involved a retrospective case-control analysis of patients diagnosed with progressive keratoconus who underwent CXL procedure in Taiwan between 2021 and 2023. Comprehensive evaluations, including slit-lamp biomicroscopy and corneal tomography, were performed at all follow-up visits. The follow-up examinations were conducted from 12 through 31 months postprocedure.

This study was approved by the Institutional Review Board (IRB) of Shin Kong Wu Ho-Su Memorial Hospital (IRB no. 20240403R) and was conducted according to the tenets of the Declaration of Helsinki (1964).

### 2.2. Participants

Twenty-four participants (totaling 31 eyes) were included in this study. The inclusion criteria required individuals diagnosed with progressive keratoconus, defined by an increase of 1 diopter (D) or more in the steepest keratometry reading, or documented historical progression (such as changes in corrective glasses, visual acuity, or contact lenses). Participants were also required to be at least 18 years old, willing and able to attend scheduled follow-up appointments, and have a minimum corneal thickness of 400 μm as measured by tomography. Exclusion criteria included prior corneal surgeries (such as intrastromal ring segments, corneal transplantation, or refractive surgeries), a history of ocular diseases that could predispose the eye to complications, clinically significant scarring within the CXL treatment zone, retinal or optic nerve disorders affecting visual acuity, or conditions considered by the surgeon to be too advanced for the procedure to be beneficial. This retrospective study was conducted in accordance with the tenets of the Declaration of Helsinki (1964) and was approved by the IRB of Shin Kong Wu Ho-Su Memorial Hospital (IRB no. 20240403R). Given the retrospective nature of this study, the IRB waived the requirement for obtaining written informed consent. All patient data were anonymized and handled with strict confidentiality throughout the research process.

### 2.3. Assessment

During the preoperative evaluation and at 1, 3, 6, and 12 months after CXL, assessments were performed. After the first year, follow-up evaluations were conducted at regular intervals of 3–6 months. The assessments included a Snellen test to measure corrected distance visual acuity (CDVA), manifest refraction, slit-lamp biomicroscopy, and corneal tomography (Pentacam, Oculus GmbH, Wetzlar, Germany). Up to three corneal tomography scans were performed for each patient to ensure diagnostic accuracy by adhering to the established quality standards. Corneal densitometry was obtained from the Pentacam's Corneal Density Map. Four concentric zones centered on the corneal apex (0–2, 2–6, 6–10, and 10–12 mm) and three layers (anterior 120 μm, central layer, and posterior 60 μm) were analyzed. We excluded the 10–12 mm zone from our main comparison because it is typically less affected by the CXL procedure. For each eye, the corneal densitometry data were recorded in grayscale units (GSUs), where 0 indicates minimal light scattering and 100 indicates maximal light scattering. In addition, the depth of the demarcation line was measured at the 1-month follow-up using anterior segment corneal spectral domain optical coherence tomography (AS-OCT) (RTVue XR-100, OptoVue, Freemont, Irvine, California, United States of America).

### 2.4. Surgical Procedure

The CXL procedure was performed in an operating room that adhered to standard surgical specifications. After disinfecting and isolating the surgical area with a sterile drape, the patient's eyelashes were taped, and a lid speculum was used to keep the eye open throughout the procedure. The corneal epithelium was manually debrided over a 9 mm diameter area using a swab moistened with 20% alcohol to facilitate the process. Riboflavin 0.25% with dextran solution (Ribotec, Implantech, South America., Argentina) was then applied topically to the cornea every 2 min. The protocol ensured thorough corneal stroma penetration and saturation, with the final riboflavin application occurring 15 min after the initial soak. Excess riboflavin was gently rinsed off using a balanced salt solution. UVA light exposure was administered using the CCL VARIO system (MLase AG, Inc., Germany), with the treated area receiving 365 nm wavelength light. The parameters for UVA exposure included a UVA irradiance of 18 mW/cm^2^, an exposure time of 5 min, and a total energy delivery of 5.4 J/cm^2^ to the corneal surface. During UVA irradiation, we applied 1-2 drops of riboflavin every 2 min for a total of two applications (at 2 and 4 minutes). This step helps maintain adequate stromal saturation, ensures uniform riboflavin distribution, and may protect deeper tissues throughout the procedure [21].

### 2.5. Statistical Analysis

To address potential intereye correlation bias arising from the inclusion of both eyes from some participants, we performed a sensitivity analysis. For participants with bilateral involvement (7 eyes), only the eye with the highest baseline *K*_max_ was included in the analysis. The results of the sensitivity analysis were compared with those of the primary analysis to evaluate the reliability of the findings. We concluded that intereye correlation had a minimal influence on the observed trends or conclusions.

Anonymized data were analyzed using SPSS Statistics (IBM Corp., Armonk, New York, United States of America). Descriptive statistics are reported as mean ± standard deviation (SD). An elevated densitometry value was defined as an increase of 1.0 GSU, based on a 3% average repeatability for the overall corneal densitometry assessment. The duration of haze was calculated as the time from the date of cross-linking to the date when corneal densitometry returned to the preoperative value (within ±1.0 GSU) [22]. Persistent corneal haze was defined as elevated densitometry values persisting at the last follow-up visit. For patients lost to follow-up, the duration of haze was calculated as the time from cross-linking to the last recorded visit. Corneal densitometry survival following cross-linking was analyzed using the Kaplan–Meier method. Differences in survival across groups, stratified by factors such as sex, median age, median preoperative *K*_max_, CCT, and median DLD at the 1-month follow-up, were assessed using the log-rank test. Patients were divided into subgroups based on the median values of age, *K*_max_, CCT, and DLD. For example, those younger than 24 years were grouped as “younger,” and those 24 years and older as “older.” The medians of *K*_max_, CCT, and DLD were 58D, 480 μm, and 392 μm, respectively. The Wilcoxon signed-rank test was employed to compare variables (corneal densitometry, *K*_max_, and CCT) before cross-linking and after the resolution of corneal haze, as the Shapiro–Wilk test indicated a non-normal distribution. A *p* value of less than 0.05 was considered statistically significant.

## 3. Results

A total of 31 eyes were included in this study. The mean age of the patients was 25.74 ± 4.78 years, with a median age of 24 years. The gender distribution was equal, with 12 males and 12 females. The mean values for *K*_max_, CCT, and DLD were 61.02 ± 9.02 D, 485.35 ± 29.18 micrometers (μm), and 373.87 ± 54.49 μm, respectively (Table 1).

Changes in corneal densitometry following cross-linking were observed across a 1-month follow-up period, as shown in Table 2.

A significant increase in densitometry values at 1-month postoperation was found across all corneal layers and zones (Φ 0–2 mm, Φ 2–6 mm, and Φ 6–10 mm), except in the posterior cornea within the 6–10 mm zone. These values then gradually decreased over time, with most regions approaching baseline levels by 12 months postoperation. At this point, the densitometry values showed no significant difference from the preoperation values.

The correlation analysis between corneal densitometry in the Φ 0–2 mm corneal zone 1 month after the procedure and DLD revealed that there was no significant correlation between the two variables across different corneal layers. The correlation coefficients were −0.006 (*p*=0.753) for the total cornea, −0.021 (*p*=0.523) for the anterior cornea, and 0.01 (*p*=0.291) for the posterior cornea, indicating that DLD did not directly affect the densitometry values in any specific corneal layer (Table 3).

In addition, we evaluated correlations between total corneal densitometry in the Φ 0–2 mm zone and both visual acuity and *K*_max_ at 1, 3, 6, and 12 months postoperatively. A transient positive association between total corneal densitometry and *K*_max_ at 1 month post-CXL (*r* = 0.47, *p*=0.01) was not observed at later follow-up points. No significant correlation was found with CDVA at any time (all *p* > 0.05).

The time to clarity (Table 4), defined as the duration required for densitometry values to return to within the baseline range, varied depending on the corneal layer and was further analyzed according to gender, age, *K*_max_, CCT, and DLD. First, the densitometry of the total cornea returned to baseline at 13.39 months.

Further analysis within subgroups showed that younger patients demonstrated a significantly faster recovery, with a mean time to clarity of 11.41 months (*p*=0.02) compared to older patients (age ≥ 24 years). Similarly, patients with a shallower DLD required a longer time to achieve clarity (14.92 months, *p*=0.046) compared to those with deeper DLD (> 392 μm). This trend was observed not only in the total cornea but also across different corneal layers. Gender, *K*_max_, and CCT did not significantly impact the time to clarity, as their associated *p* values indicated no significant differences.

## 4. Discussion

Densitometry is a quantitative method of assessing corneal clarity, which can provide a more objective perspective on the healing process after CXL. Immediately following CXL, corneal structural alterations can manifest as “haze,” and densitometry values typically increase due to the changes in collagen architecture and keratocyte population. Studies have shown that densitometry typically increases shortly after surgery due to the development of haze, as part of the corneal healing process [5]. As the post-CXL cornea remodels and heals, these values gradually return to baseline, indicating the resolution of haze and recovery of corneal transparency [6]. Thus, the recovery reflects the overall healing process after CXL.

The baseline demographic and clinical characteristics are presented in Table 1. A relatively young sample group with progressive keratoconus is indicated by the mean age of 25.74 ± 4.78 years and the advanced disease stages, as reflected in the *K*_max_ and CCT values. Prior studies have found that younger age and more advanced keratoconus can complicate procedure outcomes, particularly in long-term disease stabilization [5, 6]. In previous studies, changes in corneal densitometry after conventional cross-linking (CCXL) have been shown to be influenced by patient age. Pircher et al. [23] reported that densitometry values in the 0.0–2.0 mm zone stayed significantly elevated after CCXL (3 mW/cm^2^) for progressive keratoconus. Similarly, Greenstein et al. [24] observed a significant increase in densitometry following the same protocol. The mean ± SD age of patients in these studies was 30 ± 11 years and 32 ± 10 years, respectively. Conversely, Alnawaiseh et al. [25], with a younger patient group (mean age 27.9 ± 8.6 years), found that densitometry returned to preoperative levels at 12 months post-CCXL. In addition, Kim et al. [9] reported a similar recovery in densitometry by 6 months post-CCXL in even younger patients (mean age: 21.9 ± 6.1 years). These findings suggest that age may play a critical role in the recovery of corneal transparency after CXL, with younger patients demonstrating a faster return to baseline densitometry values. Our results are consistent with previous studies. Table 4 shows that the group with a mean age of 22.3 ± 1.7 years had a significantly shorter recovery time than the group with a mean age of 29.5 ± 4.1 years.

Since the introduction of accelerated CXL, various protocols with different irradiation patterns have been developed [2]. Although these protocols theoretically achieve the same total energy, they may still result in different outcomes. Our protocol, which uses epi-off and 0.25% riboflavin with an irradiation of 18 mW/cm^2^ for 5 min (total dose of 5.4 J), is rarely reported in the literature compared to other accelerated CXL protocols. In Table 2, excluding the 6–10 mm zone, which appears to be less impacted by the CXL effect due to its peripheral location, we observed that, regardless of the total cornea or other layers, clarity was restored by 12 months postprocedure. In other words, the densitometry values showed no significant difference when compared to preoperation levels. A study on transepithelial CXL with 0.1% riboflavin and 3 mW/cm^2^ irradiation for 30 min reported that densitometry values returned to baseline within 12 months postprocedure [5]. Another study using a high-intensity protocol (0.25% riboflavin and 45 mW/cm^2^ in pulsed mode for a total dose of 7.2 J/cm^2^) observed a significantly longer recovery time of 24 months, likely due to the higher total energy applied to the cornea [6]. This suggests that the total energy delivered during CXL could be a critical factor influencing the recovery of corneal clarity. In contrast, Chan et al. [8] employing an accelerated CXL protocol similar to ours (0.1% riboflavin, epi-off, and 18 mW/cm^2^ for 5 min) found that the densitometry values in the 0–2 mm corneal zone recovered within 18.2 ± 3.8 months, and in the 2–6 mm zone, within 10.9 ± 2.5 months. This recovery period appears slightly longer than ours, possibly due to the difference in patient demographics, as their sample group had an older mean age of 28.8 ± 7.5 years compared to our younger sample group. In addition, a study [4] comparing three different CXL protocols, including standard (30 min, 3 mW/cm^2^), transepithelial (30 min, 5.4 J/cm^2^), and accelerated (9 min, 5.4 J/cm^2^) protocols, found varying recovery times. In the standard protocol, densitometry returned to baseline around 12 months, while the transepithelial protocol showed a faster recovery at 6 months. The accelerated protocol resulted in densitometry values that were still higher than baseline at 12 months, indicating a slower recovery process. Our protocol demonstrated a recovery time that aligns well with these different findings, suggesting that this high-concentration, short-duration protocol can be both effective and relatively safe. The potential benefits include reduced treatment duration and possibly deeper or more uniform cross-linking; however, any increased UVA shielding or risk of deeper tissue damage at higher riboflavin concentrations must be monitored. Although we observed no significant adverse events within our 12-month follow-up, more comprehensive and longer-term studies remain essential to fully assess this approach. In addition, we have conducted a separate analysis focusing on the impact of 0.25% riboflavin on visual acuity and keratometric parameters (*K*_max_) in the same sample group (manuscript currently under review). While the present paper centers on corneal densitometry and clarity recovery, the complementary data from the separate analysis further elucidates the clinical effects of this high-concentration, accelerated regimen on visual function and corneal topography.

Correlation analysis in Table 3 reveals no significant association between DLD and densitometry value measured in the Φ 0–2 mm corneal zone, which is the region most affected by the CXL procedure. This lack of correlation may be explained by the fact that DLD reflects biomechanical effects rather than optical outcomes such as ttransparency. However, a clinical study [17] revealed that dynamic changes in corneal biomechanics following CXL do not appear to be associated with DLD. Furthermore, Hafezi et al. have shown that variations in DLD, even across different CXL protocols, do not consistently reflect changes in optical clarity [26]. This suggests that while DLD indicates the extent of the CXL effect, it does not directly influence postoperative corneal transparency. Interestingly, when exploring the factors affecting healing time, we were surprised to find that DLD showed a significant difference (Table 4). This significance was observed in both the 0–2 mm and 2–6 mm annular zones of the cornea, with a similar trend found in other zones. Densitometry values for cornea with deeper DLD recovered densitometry values to baseline faster, within 7–11 months, whereas corneas with shallower DLD took approximately 12–15 months. While deeper cross-linking may not directly influence immediate postoperative transparency, it might contribute to a more efficient corneal remodeling process over time. The concept of the stromal DLD as an indicator of CXL procedure efficacy was initially proposed by Seiler and Hafezi [16] suggesting that DLD could serve as a marker to verify the depth of the CXL effect, providing insight into the extent of corneal stromal cross-linking. Subsequent studies have further explored this idea, with some findings supporting the view that a deeper DLD correlates with more extensive cross-linking and potentially enhanced biomechanical strengthening of the cornea [27, 28]. However, the assumption that a deeper DLD directly translates to better clinical outcomes remains controversial. Several reviews have suggested that while DLD offers a valuable means to assess the extent of the cross-linked corneal region, its direct correlation with visual and optical outcomes, such as corneal transparency, is not consistently observed [17, 29–31]. One possible explanation for the faster recovery of densitometry values in the deeper DLD group could be related to the distribution and intensity of the CXL effect. Shallower DLDs might indicate a more superficial and potentially concentrated cross-linking effect, leading to a slower resolution of haze and delayed restoration of corneal transparency. Indeed, we observed a trend toward higher densitometry values at 1-month postprocedure in the shallow DLD group, supporting this hypothesis. Future studies are required to confirm these findings. Ultimately, while DLD is a useful marker for cross-linking extent, our findings suggest that its depth does not directly predict corneal clarity but may influence overall recovery time.

This study has several limitations. First, the small sample size and retrospective design limit the generalizability of our findings. Second, the varied follow-up duration among participants may introduce bias in assessing long-term recovery. Third, interobserver variability in measuring DLD using AS-OCT could affect the consistency of our data. In addition, our focus on densitometry alone does not capture other aspects of corneal healing, such as biomechanical properties and visual acuity. We also lacked a separate control group (e.g., 0.1% riboflavin or no treatment group) for direct comparison, which constrains our ability to draw definitive conclusions about protocol superiority. Lastly, the findings may not be directly applicable to other CXL protocols, as this study only examined a specific accelerated protocol using 0.25% riboflavin and 18 mW/cm^2^ for 5 min.

## 5. Conclusion

We demonstrate that corneal densitometry is a reliable measure of the healing process following CXL, particularly in assessing the resolution of postoperative haze. Deeper DLDs and younger ages are associated with a faster return to baseline densitometry values, indicating a potentially more efficient corneal remodeling process. Although DLD depth does not directly predict corneal clarity, it may influence the overall recovery time. Moreover, with the application of 0.25% riboflavin for a short-duration, high-irradiance protocol appears safe and effective, with most patients recovering corneal clarity within 12 months.

## Figures and Tables

**Table 1:** Demographics and keratometric measurements before cross-linking.

Total eyes (*N* = 31)	*N* (%)/mean ± SD	Median
Age (years)	25.74 ± 4.78	24
Sex		
Male	12 (50%)	
Female	12 (50%)	
*K*_max_ (D)	61.02 ± 9.02	61.4
CCT (μm)	485.35 ± 29.18	483.5
DLD (μm)	373.87 ± 54.49	392

*Note:K*_max_ = maximum anterior keratometry radius.

Abbreviations: CCT = central corneal thickness; DLD = demarcation line depth.

**Table 2:** Corneal densitometry changes after cross-linking.

*N* = 31 (mean ± SD)	Preoperation	Month 1	Month 3	Month 6	Month 12
*Total*	15.3 ± 2.2	19.3 ± 2.5	18.1 ± 2.4	16.3 ± 2.3	**15.3 ± 2.1**
Φ 0–2 mm	16.0 ± 2.3	24.1 ± 5.1	23.1 ± 5.6	18.9 ± 4.7	**16.8 ± 3.5**
Φ 2–6 mm	14.8 ± 1.6	21.0 ± 2.5	18.7 ± 2.5	16.0 ± 1.7	**14.8 ± 2.0**
Φ 6–10 mm	13.3 ± 2.6	14.7 ± 2.5	14.0 ± 2.4	13.3 ± 2.3	12.8 ± 2.2

*Anterior*	21.3 ± 3.4	28.6 ± 4.1	26.5 ± 3.7	23.2 ± 3.5	**21.3 ± 3.3**
Φ 0–2 mm	23.6 ± 4.2	38.1 ± 9.0	36.1 ± 11.6	28.7 ± 8.6	**24.8 ± 5.9**
Φ 2–6 mm	21.0 ± 2.3	32.1 ± 4.6	28.2 ± 3.7	23.1 ± 2.4	**21.1 ± 3.1**
Φ 6–10 mm	17.5 ± 3.8	20.7 ± 3.6	19.1 ± 3.7	17.9 ± 3.6	16.9 ± 3.3

*Central*	14.1 ± 1.9	17.4 ± 2.3	16.2 ± 2.5	14.9 ± 2.2	**13.9 ± 1.8**
Φ 0–2 mm	15.4 ± 1.6	22.4 ± 4.9	21.3 ± 5.6	18.1 ± 4.4	**16.1 ± 2.8**
Φ 2–6 mm	13.3 ± 1.2	18.7 ± 2.2	16.2 ± 2.6	14.3 ± 1.8	**13.1 ± 1.6**
Φ 6–10 mm	12.3 ± 2.2	13.1 ± 2.3	**12.7 ± 2.2**	**12.1 ± 2.0**	**11.7 ± 1.9**

*Posterior*	10.6 ± 1.6	11.8 ± 1.6	11.6 ± 1.6	**10.8 ± 1.4**	**10.6 ± 1.4**
Φ 0–2 mm	8.9 ± 1.8	11.7 ± 2.8	11.2 ± 2.8	**9.7 ± 2.2**	**9.5 ± 1.9**
Φ 2–6 mm	10.2 ± 1.7	12.7 ± 2.3	11.7 ± 2.3	**10.5 ± 1.8**	**10.3 ± 1.6**
Φ 6–10 mm	9.9 ± 1.9	**10.2 ± 1.8**	**10.3 ± 1.5**	**9.8 ± 1.4**	**9.8 ± 1.6**

*Note:* Φ: Annular diameters. Bold value indicates “no” significant difference in densitometry values between preoperation and postoperation.

**Table 3:** Correlation analysis of densitometry value and demarcation line depth.

Total eyes (*N* = 31)	Coefficient	*p* value
Total cornea	−0.006	0.753
Anterior cornea	−0.021	0.523
Posterior cornea	0.01	0.291

*Note:* Densitometry values were measured in the Φ 0–2 mm corneal zone. The analysis included correlations with DLD at 1-month post-CXL across different corneal layers (anterior, central, and posterior).

**Table 4:** Time to clarity and correlation analysis across different corneal layers.

Total	13.39 months			
	Φ 0–2 mm		Φ 2–6 mm	
	Time to clarity	*p* value	Time to clarity	*p* value
*Gender*				
Male	13.67	0.66	11.39	0.31
Female	12.85		8.23	

*Age*				
Younger	11.41	0.02⁣^∗^	8.59	0.13
Older	15.79		11.96	

*K*_max_				
Flat	14.38	0.39	10.38	0.72
Steep	12.20		9.80	

*CCT*				
Thin	14.38	0.24	12.75	0.03⁣^∗^
Thick	12.20		7.07	

*DLD*				
Shallow	14.92	0.046⁣^∗^	11.92	0.03⁣^∗^
Deep	11.39		7.00	

*Note:* Gender was divided into male and female groups. For all other variables (Age, *K*_max_, CCT, and DLD), Group 1 and Group 2 were determined based on whether values were below or above the median (see Table 1).

“^∗^” indicates a statistically significant difference with *p* < 0.05.

## Data Availability

The data that support the findings of this study are available from the corresponding author upon reasonable request.
